# Arterial Vascularization of the Forehead in Aesthetic Dermatology Procedures: A Review

**DOI:** 10.3390/jcm13144238

**Published:** 2024-07-19

**Authors:** Katarzyna Kliniec, Zygmunt Domagała, Bartosz Kempisty, Jacek C. Szepietowski

**Affiliations:** 1 Department of Dermatology, Venereology and Allergology, Wroclaw Medical University, 50-368 Wroclaw, Poland; katarzyna.kliniec@gmail.com; 2 Division of Anatomy, Department of Human Morphology and Embryology, Wroclaw Medical University, 50-368 Wroclaw, Poland; zygmunt.domagala@umw.edu.pl (Z.D.); bartosz.kempisty@umw.edu.pl (B.K.); 3 Institute of Veterinary Medicine, Nicolaus Copernicus University, 87-100 Torun, Poland; 4 Physiology Graduate Faculty, North Carolina State University, Raleigh, NC 27695, USA; 5 Center of Assisted Reproduction, Department of Obstetrics and Gynecology, University Hospital and Masaryk University, 625 00 Brno, Czech Republic

**Keywords:** frontal arteries, aesthetic procedures, dermatology, glabella, central forehead, supraorbital artery, supratrochlear artery, paracentral artery, central artery, frontal branch of superficial temporal artery

## Abstract

**Background:** The growing popularity of aesthetic procedures on the face raises the question of their safety. The forehead region is crucial aesthetically, but due to its abundant vascularization, it is also one of the most dangerous areas for dermatologic procedures, especially in the glabella area. The purpose of this article is to review the literature on the arterial vascularization of the forehead to identify potential high-risk zones for aesthetic dermatology procedures. **Methods:** A database search (PubMed, Web of Science, Scopus, and Embase) was conducted, and the titles and abstracts of all identified studies were screened, followed by full-text evaluation. **Results:** We identified 714 articles during the database search, and 25 articles were included in the review. The included studies used cadaveric dissection and computed tomography applied to cadavers as well as Doppler ultrasonography on volunteers to evaluate the forehead arteries (supratrochlear (STrA), supraorbital (SOA), central (CA), paracentral artery (PCA), and frontal branch of superficial temporal artery(FBSTA)). A total of 1714 cases involving the forehead arteries were analyzed. The included arteries were observed over a relatively large area, and their locations varied. The CA and PCA in cadaver studies were observed in an area of 0.2 to 10.8 mm and 0.8 to 16.2 mm, respectively, on the entire path from the glabellar point to the frontal prominence point. The distances from the midline in cadaveric studies at various measurement points ranged from 0.6 to 28.0 mm for the superficial branch of the STrA and 13.6 to 40.7 mm for the deep branch of STrA. In case of SOA, the distance from the midline ranged from 23 to 32 mm. Measurements from the midline in Doppler studies ranged from 0 to 23 mm for STrA and from 10 to 50 mm for the SOA. In studies using computed tomography, STrA was observed at a distance of 11 to 21 mm and the SOA at a distance of 21 to 32 mm, both lateral to the midline. **Conclusions:** Medical professionals should be aware of zones where frontal arteries are more likely to be encountered. The glabella region appears to be one of the most dangerous areas for dermatologic procedures. It is believed that the supratrochlear, supraorbital, and the paracentral arteries may cause ophthalmic complications due to occlusion of the ophthalmic artery, while this risk for the frontal branch of the superficial temporal artery seems to be low but cannot be completely excluded.

## 1. Introduction

In recent years, the popularity of aesthetic and dermatological procedures is constantly growing [1,2,3]. Treatments like fillers (e.g., hyaluronic acid) or autologous fat injections are performed not only for anti-aging measures like wrinkle correction but are also applied in cases of serious aesthetic complications of certain dermatological diseases, such as progressive facial hemi-atrophy (Parry–Romberg syndrome), acne scarring, or lupus [2,3,4,5,6,7,8]. The alarming trend of a growing numbers of people undertaking such procedures without the necessary skills or qualifications is a cause for concern, especially because of the serious complications that may occur [1]. The most serious of these involve vascular complications [2,8].

The forehead has a crucial importance in aesthetic aspects and is one of the most common areas for dermatological procedures [9]. However, it is also one of the most dangerous areas for such interventions, especially the central forehead with its glabella zone [3,10]. This can be attributed to the abundant vascularization of this area and the increasing popularity of treatments in this particular region [3,9]. In the forehead, blood supply is primarily provided by arteries such as the supratrochlear artery (STrA), supraorbital artery (SOA), central artery (CA), paracentral artery(PCA), or frontal branch of the superficial temporal artery (FBSTA) [9]. Material applied into these arteries and subsequent occlusion can lead to severe complications like visual disturbances, including blindness, skin necrosis, or stroke [4,9]. Moreover, the individual variability of the aforementioned arteries makes these procedures even more challenging [4,11]. To provide safety, professionals must possess a thorough understanding of the vascular anatomy specific to the treatment area, be aware of high-risk zones for vascular complications, and employ techniques such as ultrasonography to enhance safety during the procedures [4,11]. The current review aimed to analyze the literature findings on the arterial vascularization of the forehead to provide insight into the variability of these vessels and to identify potential high-risk zones for aesthetic dermatology procedures.

## 2. Materials and Methods

The study was conducted according to 2020 Preferred Reported Items for Systematic Reviews and Meta-Analyses (PRISMA) guidelines [12]. Prior to the conduct of this review, a protocol that adhered to the PRISMA-protocols guidelines was developed and then registered on International Platform of Registered Systematic Review and Meta-analysis Protocols (INPLASY)—(https://inplasy.com/inplasy-2024-6-0099/ accessed 25 June 2024).

Database research was performed between February 2024 and April 2024, including PubMed, Web of Science, Scopus, and Embase records. The search terms are presented in the Supplementary Materials (Supplementary Material File S1). The search results were restricted to studies published from 2004 to 2024. The results were exported from the databases to the citation manager—EndNote 21 (Clarivate, Philadelphia, PA, USA). Duplicate records were excluded. Inclusion criteria were articles in English; anatomical studies concerning the course, location, and variations of the frontal arteries (supratrochlear, supraorbital, central, paracentral artery, and frontal branch of superficial temporal artery); and articles within the context of safety in dermatological, dermatosurgical, and aesthetic procedures. This includes studies employing various methods of arterial evaluation, such as Doppler ultrasonography, computed tomography, and cadaver dissection. Exclusion criteria included no English version of the article; lack of full text availability; studies on dermatological, dermatosurgical, and aesthetic procedures specific to the forehead but unrelated to the anatomy of forehead arteries; studies focused on different facial areas; and reviews, commentary, letters to the editor, and conference abstracts.

**Figure 1:**
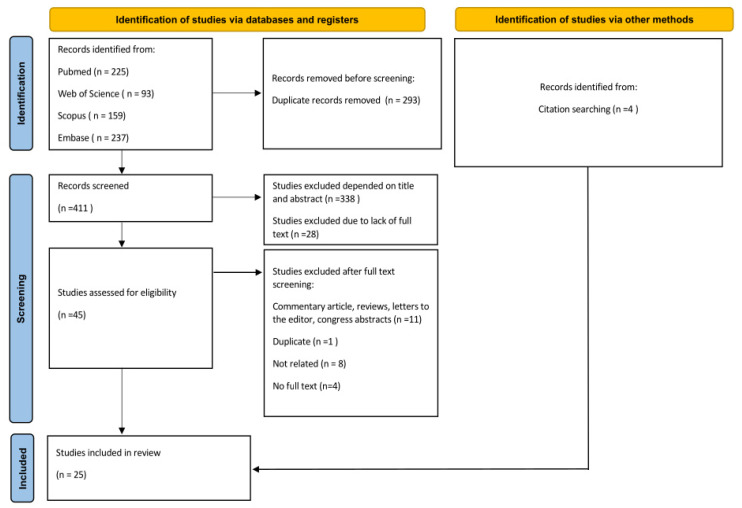
PRISMA flow diagram for included studies.

The titles and abstracts of all identified studies were screened independently by two authors (K.K. and J.C.S.). The full texts of the studies that could not be determined to meet the inclusion criteria were examined. The initial screening was followed by a full-text evaluation. All differences were resolved through consultation among the two authors (K.K. and J.C.S.). In addition, the references list of included studies was examined to identify any not yet included studies that met inclusion criteria. Details of the study selection process are shown in Figure 1.

The following information was recorded from these articles: first author, date of publication, number of group, gender of patients, age of patients, arteries covered, method of arterial evaluation, and outcomes (diameter, measure points, distance from landmarks, and branching of the arteries) For a more accessible presentation of the results, the extracted data are presented in tables in this paper.

The total number of cases was determined as follows. In studies where only the number of cadavers or patients was given, and measurements were taken bilaterally, the number of cases was doubled. They were then added up with cases from studies in which the number of hemifaces was given. In a few instances, cases were included based on the original number of cadavers or patients when it was uncertain whether measurements were taken on both sides of the face.

Due to the nature of the data reported in the studies included in the presented systematic review (anatomical description and measurements without specific effect changes described), no effect measures were applied in the article.

For this systematic review, no meta-analyses were conducted. Data conversion involved only minor adjustments, such as converting dimensions from centimeters to millimeters, providing consistency in certain anatomical terms if different studies used different synonyms for the name of a particular anatomical structure, and providing the number of hemifaces examined when initially not reported but feasible (e.g., when the authors disclosed the number of cadavers but examined both sides of the head) to ensure consistency of the total number of cases included. Data synthesis was performed for studies where direct comparison of the data was feasible.

No ethics committee approval was required for the literature review.

## 3. Results

Database research identified a total of 714 articles. After removing duplicates, 411 studies were screened for titles and abstracts, and the full texts were examined in the case of any doubts. Forty-five articles seemed to meet the inclusion criteria, and the full texts were reviewed. At this point, 24 studies were excluded due to inadequate study design (review, commentary, letter to the editor, or conference abstract), status as a duplicate, lack of full text, or not addressing the course and distribution of the arteries of the forehead. In addition, four articles were included through a citation search. Finally, 25 articles were included in the study.

**Figure 2:**
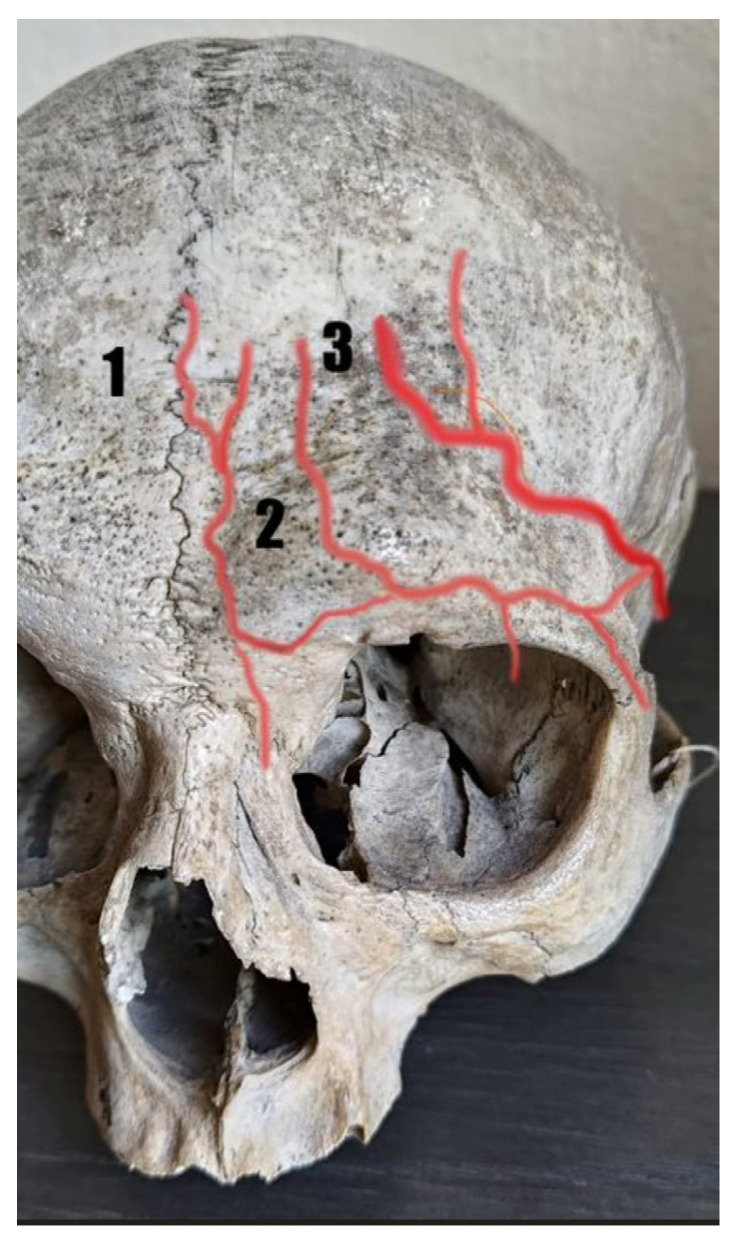
Normal anatomy of the forehead arteries: (1) supratrochlear artery, (2) supraorbital artery, and (3) frontal branch of the superficial temporal artery.

A total of 1714 cases involving the forehead arteries were analyzed. Included studies used cadaveric dissection and imaging techniques (computed tomography) applied on cadavers and Doppler ultrasonography on volunteers to evaluate the arteries. The supratrochlear artery, supraorbital artery, central artery, paracentral artery, and frontal branch of the superficial temporal artery were evaluated, including their course, variation, location in relation to landmarks, diameters, and depths. Figure 2 shows the standard anatomy of the forehead arteries. The details of studies included in the review are shown in Table 1 and Table 2.

### 3.1. Cadavers’ Dissection Studies

#### 3.1.1. Central Artery

The central artery (CA) was described in three cadaveric dissection studies [4,8,14]. It was present in 58 of 98 cases from two studies [4,14]; in one study, the authors did not provide the exact number of cases in which CA was observed, instead reporting that CA or the paracentral artery was found in 4 of 20 cases [8]. In all cases, CA was a branch of the dorsal nasal artery (DNA). The diameter of CA was determined in two studies, and the mean values were less than 1 mm [4,14] The details are shown in Table 3.

Phumyoo et al. [4] observed the CA in about 42% of cases as a branch of the DNA, arising −13.2 ± 2.7 mm vertically from glabellar point (FHL0) and 4.7 ± 1.8 mm horizontally from the midline. The artery was found to continue medially to the glabella and central forehead, located 4.7 ± 2.3 mm horizontally from FHL0, 4.4 ± 2.0 from mid-frontal depression point (FHL1), and 3.6 ± 2.6 mm from frontal prominence point (FHL2). It ran further into the subcutaneous tissue layer and became slightly more superficial in the frontal prominence area. The results are shown in Table 4. Kleintjes [14] observed CA in 42 of the hemiforehead and in all cases as a branch of the DNA. The average diameter was 1 mm. The artery provided blood to the glabella as well as the lower and middle transverse thirds of the central forehead. No other detailed measurements were described. Cong et al. [8] reported that the central or paracentral artery was observed in 20% of cases. The CA was a branch of the DNA, and it supplied the superficial part of the frontalis muscle. The authors did not provide a description of any other detailed measurements.

#### 3.1.2. Paracentral Artery

The paracentral artery (PCA) was described in four cadaveric dissection studies [4,8,14,16]. It was present in 29 of the 98 cases from two studies [4,14]. One study did not provide information on the number of cases where the PCA was observed; the authors only mentioned the radix branch of the inferior orbital artery, which was observed in 28/40 and gave rise to the PCA [16]. As described earlier, one study did not report the exact number of cases where the PCA was found, though the authors reported that the CA or the PCA was observed in 4 of 20 cases [8]. In most cases, the PCA was a branch of the angular artery. The diameter of the PCA as well as the previously mentioned CA was determined in two studies, and the mean values were less than 1 mm, as shown in Table 4 [4,14].

Phumyoo et al. [4] described the presence of a paracentral artery in slightly over 21% of subjects. It appeared as a branch of angular artery 9.4 ± 3.1 mm horizontally from the midline and −13.9 ± 4.7 mm vertically from the FHL0. It ran vertically laterally to the central artery. The horizontal distances in FHL0 and FHL1 were 7.8 ± 2.4 and 8.8 ± 2.7 mm, while in FHL2, there were significant differences in distance between men (4.1 ± 3.9 mm) and women (12.3 ± 2.4 mm). Similar to the central artery, it passed into the subcutaneous tissue layer and became more superficial in the frontal prominence area. The results are shown in Table 4. Kleintjes [14] found the paracentral artery in 21 hemiforeheads. It arises directly from the AA into the forehead (15 cases) or from the communicating branch of the AA with STrA (six cases). In Tansatit et al.’s [16] study, the paracentral artery originated from the radix branch of the inferior orbitoglabellar artery and ran in an upward direction toward the glabella. No other details of the artery were described in this article. As mentioned earlier in this article, Cong et al. [8] reported that the central artery or paracentral artery was observed in 20% of cases. The PCA was a branch of the angular artery, and it supplied the superficial part of the frontalis muscle. No other details of the artery were described in this article.

#### 3.1.3. Supratrochlear Artery

The supratrochlear artery (STrA) was described in ten cadaveric dissection studies [4,8,14,16,17,19,20,21,23,25]. The presence of STrA was estimated in eight studies, and the artery was observed in 199 of 212 cases. Two articles did not describe the number of occurrences of this vessel. In most cases, STrA appeared on the forehead near the supraorbital rim, and in a number of cases, it branched deeply relative to the corrugators supercilii muscle.

The superficial branch of STrA was described in three studies and was present in 63 of 68 cases [4,8]. It usually continued to eventually become superficial to the frontalis muscle. A deep branch was described in three studies and observed in 35 of 68 cases [4,8]. In a number of cases, it was noted to run deep to the frontalis muscle. The distance of STrA from the midline was determined in three studies on cadavers [4,19,25]. It was measured at several points, and the results were presented for the STrA or separately for the superficial and deep branches of the STrA, depending on the study. The detailed results of the aforementioned studies are presented further in the article and in Table 4. Three studies described the location of the STrA in relation to the medial canthus (MC) [8,14,20]. It mostly did not deviate by more than 5 mm from MC. However, in regard to the most common location, one study reported the deep branch runs mainly laterally, while another study, which did not distinguish between branches, found the STrA was predominantly located medially to MC [8,14]. Two studies examined the vertical distance from the supraorbital rim where the artery became subcutaneous [14,23]. According to reports from one study, this distance ranged from 15 to 25 mm, while another study found it to be about 15 mm above the supraorbital rim. The diameter of the STrA was defined in three studies. The average diameter ranged from 0.6 to 1.08 mm, depending on the study, the branch, and the point of measurement; details are shown in Table 3.

Phumyoo et al. [4] found a superficial branch of the STrA in 86.84% of subjects and a deep branch in more than 21% of subjects. Both branches arise deeply from the corrugators supercilii muscle, near the supraorbital rim and the level of the glabellar point. In most cases, there was a single superficial branch, while in 36.84% of the subjects, the authors observed a double superficial branch, which represented a medial and lateral segment. At the glabellar point, the distance of the superficial branch from the midline was 14.7 ± 3.2; further, the branch crossed the corrugator supercilii muscle and, 13.9 ± 3.3 mm horizontally from the midline and 7.6 ± 2.6 mm vertically from the FHL0, penetrated the frontalis muscle into the subcutaneous layer. It deviated medially of the central forehead 12.2 ± 3.1 mm horizontally at the level of FHL1 and 11.7 ± 4.2 mm horizontally at the level of FHL2. The deep branch ran in the intramuscular or periosteal layer. The distance from the midline at the FHL0 level was 19.2 ± 1.6 mm. It then penetrated the corrugator muscle or ran deeply into it, and above FHL1, it penetrated the frontalis muscle. The vertical distance from FHL0 was 30.2 ± 5.1 mm, and the horizontal distance from the midline was 25.5 ± 5.6 mm. To the midline of the forehead, the deep branch deviated laterally. In FHL1, the horizontal distance was 21.4 ± 3.9 mm, and in FHL2, it was 27.2 ± 6.1 mm. Tansatit et al. [16] found that the branch of the ophthalmic artery—the superior orbitoglabellar artery—divided into the supratrochlear artery and the supraorbital artery near the supraorbital rim in 15 of 40 cadavers’ sides included in the study. The STrA then ran vertically to the medial eyebrow and on to the frontal hairline. In 12.5% of cases, the STrA diverged directly from another branch of ophthalmic artery, i.e., the inferior orbitoglabellar artery, and in 15% of cases, it arose from the radix branch of the inferior orbitoglabellar artery and continued laterally from the midline. In the study conducted by Khan et al. [17] concerning the STrA, there was no information given on its run. The authors measured the length, radius, and the volume of the STrA. The average length was 51.75 ± 9.98 mm, radius 0.72 ± 0.08 mm, and the volume 0.085 ± 0.02 mL. Cong et al. [8] described the STrA to appear at the lower medial margin of the orbital rim in 85% of the cases. It continued ascending along the frontalis muscle, laterally to the midline. It divided into superficial and deep branches that provide blood to the frontalis muscle. The superficial branches of the STrA were seen in 100% of the cases and ran superficially to the frontalis muscle. The deep branch was reported in 55% of the cases and mostly was located laterally to the medial canthus plane. In the Kleintjes [14] study, the STrA was found in 58 cases. It was noted to originate from the superomedial orbit and mostly did not deviate more than 5 mm from medial canthal vertical line in the inferior transverse third. It was located medial to this line. The STrA perforated the corrugator supercilii muscle, and 15–25 mm above the supraorbital rim (SOR), it appeared subcutaneous. It ran superficially: 1–2 mm deep in the muscle layers. In 23 cases, the STrA had medial and lateral branches that typically originated at the inferior and middle third junction; in 20 cases, there was only one branch. The branches were mostly observed to run superiorly from the level of the middle transverse third towards the midline. Multiple branches of STrA were described, the most common being the medial communicating branch (MCB) with the AA at the SOR (60%). Yu et al. [19] observed that the STrA ran over the corrugator muscle and under the orbicularis oculi muscle and progressively became superficial. The cutaneous branch of STrA originated 11.8 ± 3.6 mm distal to the supraorbital rim and 13.5 ± 3.4 mm lateral to the midline in seven of the cases. The position of the branch on the body surface was in the medial part of the eyebrow. The branch continued superomedially and ran subcutaneously almost its entire course. The superior 1/3 ran beneath the dermis and above the fat tissue, and the inferior 2/3 ran above the frontalis muscle beneath the fat tissue. The terminal (muscular) branch of the STrA ran under the frontalis muscle. In three specimens, the STrA ran subcutaneously under the medial aspect of the brow and became a cutaneous branch as a direct extension. No muscular branch was observed. Palomar-Gallego et al. [21] followed the course of the supratrochlear artery as it originated from the orbit 11.5 (8–13.5) mm above the medial palpebral commissure and ran over the procerus muscle before reaching the glabella. In the study by Ugur et al. [20], the StrA was found in 14 of 20 sides of cadavers. The authors described the location and distance according to the medical canthus, and it ranged from 0 to 3 mm. The detailed results are shown in Table 4. Reece et al. [23] followed the supratrochlear vessel as it left the medial orbit, travelling on the periosteum and then branching before reaching the corrugator muscle, giving a periosteal and superficial branch. Above the division, the superficial branch was located between the corrugator supercilli and orbicularis oculi muscles as it ascended the forehead. In the further course, about 15 mm above supraorbital rim, it was positioned subcutaneously, superficially to the frontalis muscle. Then, it maintained its position consistently up to 40 mm above the supraorbital rim. Schwenn et al. [25] noted that the STrA tended to give its terminal branches while lying superficially to the orbicularis oculi muscle in the subcutaneous space. The authors reported the distance from the midline at the supraorbital rim, and it ranged from 14 to 21 mm. The diameter varied between 0.8 and 1.5 mm.

#### 3.1.4. Supraorbital Artery

The supraorbital artery (SOA) was described in six cadaveric dissection studies [8,14,16,18,21,25]. The presence of SOA was estimated in five of them, and the artery was seen in 169 of 170 cases [8,14,16,18,25]. One article did not specify the number of observed cases in which this artery was present [21]. In 85 of 101 cases, the SOA was described to pass over the supraorbital rim or to enter the forehead via supraorbital notch, and in 16 cases, it ran through a foramen. Mostly, it was divided into superficial and deep branches. In most cases, it was described to perforate the frontalis muscle to continue superficially. The penetration point was determined in two studies, with an average distance of 20.7 mm determined in one study and a range of 20 to 40 mm from the level corresponding to the supraorbital rim established in the other study [8,18]. In two studies, the distance from the midline was noted and ranged from 23 to 32 mm, measured at the point where the SOA left the orbit or at the level of supraorbital rim [21,25]. The diameter of the SOA was established in two studies. Average values ranged from 0.86 to 1 mm [14,25].

The supraorbital artery (SOA) was observed in 60 hemiforeheads in the Kleintjes [14] study. In 58 of them, the SOA passed over the supraorbital rim and in two hemiforeheads went through a foramen. The diameter was 1 mm. In the vertical plane, it ran through the medial limbus of the cornea, arising over supraorbital rim and then heading laterally. The branches of SOA were identified in 76.67% of the subjects, including a lateral rim branch (39/46), oblique branch, (39/46), medial branch (19/46), vertical branch (43/46), and brow branch (2/46). The first three always had a deep (periosteal) course; the vertical branch usually ran periosteally and the brow branch superficially and laterally. As mentioned earlier, in the study by Tansatit et al. [16], the SOA was found to be one of the branches of the superior orbitoglabellar artery or branched directly from the ophthalmic artery. In the latter scenario, in 25% of cases, it emerged from the supraorbital notch and in 10% from supraorbital foramen. It was divided into vertical, oblique, and transverse branches near the supraorbital foramen. In the study by Cong et al. [8], the SOA emerged at the lower margin of the orbital rim from the supraorbital foramen or notch. The foramen was observed 10.8 ± 4.9 mm horizontally from the vertical line running through the medial canthus (Y) and 22.1 ± 2.6 mm vertically from the horizontal line running from the medial canthus to the lateral canthus (X1). The artery continued deeply in the frontalis muscle as the deep branch of the SOA and further perforated the frontalis muscle and continued superficially as the superficial branch of the SOA. Both branches were reported in 100% of the cases. The penetration point of frontalis muscle was observed 29.6 ± 4.1 mm horizontally from Y and 20.7 ± 5.1 mm vertically from the horizontal line running through the supraorbital margin (X2). In the study by Erdogmus and Govsa [18], the SOA was reported as the terminal branch of the ophthalmic artery that ran superiorly and medially to the forehead. Its mean diameter was 0.84 mm on the right side and 0.87 mm on the left side. At the level of the supraorbital rim, the SOA entered the corrugator muscle and divided into superficial and deep branches. The authors noted that two to four superficial branches emerged from the SOA. Superficial branches continued entering the corrugator and orbicularis oculi muscles, then ran through the frontalis muscle to become superficial, and continued in the subcutaneous fat. The SOA perforated the frontalis muscle from 20 to 40 mm above the supraorbital rim. It was located in the subcutaneous tissue approximately 40 to 60 mm above the supraorbital rim. The deep branches of the supraorbital artery originated at the supraorbital rim and ran axially above the supraorbital rim for 16–42 mm. They were located in the deep layers of the subgaleal fascia just above the periosteum. The average diameter was 0.6 mm. Two patterns of origination of the deep branch were observed. Predominantly, in 79%, it arose from the SOA at the level of or below the supraorbital rim. In the remaining cases, the deep branch originated in the layer of the galea-frontalis and crossed to the pericranium 5 to 12 mm above the supraorbital rim. Palomar-Gallego et al. [21] investigated the starting point of supraorbital arteries’ superficial course and found that it left the orbit at a distance of 23 to 27 mm lateral to the glabellar midline. Schwenn et al. [25] described the course of the SOA as more variable than that of the STrA. In most (10/12) samples the authors examined, it left the orbit via the supraorbital foramen; however, in two cases, the exit point was the supraorbital notch. Looking into differences between the supratrochlear and supraorbital artery, they observed that the supraorbital artery’s outer diameter at the orbital rim was smaller than that of the supratrochlear (see Table 4). Furthermore, the supraorbital artery’s division point was located beneath the orbicularis oculi muscle, different to that of the supratrochlear artery, which bifurcates superficially to that muscle. Finally, the authors measured the distance between midline and the SOA, and it ranged between 23 and 32 mm.

#### 3.1.5. Frontal Branch of the STA

Kleintjes [14] observed the frontal branch of STA (FBSTA) in 42 hemiforeheads. It entered the forehead at various transverse levels in a vertical line through the lateral orbital rim. In 23 hemiforeheads it occurred at the junction of the inferior and middle transverse third and in five cases in the superior transverse third. It was mostly divided at or near the lateral orbital rim vertical line into an ascending and a transverse branch, which was observed in 33 cases in this study. The transverse branch was raised in the middle transverse third in 20 cases and in the superior transverse third in 13 cases. In 11 cases, there was more the one transverse branch. In the study by Pinar and Govsa [15], the FBSTA was observed in 100% of cases. The division of the STA into a frontal and parietal branch was seen above the zygomatic arch in more than 74% of cases and in about 22% just over the zygomatic arch; in one case, there was no division, only a continuation of the STA as a frontal branch. The frontal branch then ran parallel to the upper corner of the orbicularis oculi muscle to the front of the head and, reaching the frontalis muscle, it returned to the galea. In a cadaveric study by Jo et al. [13], a perforating branch of frontal branch of STA was found in 100% of hemifaces. It perforated the frontalis muscle to the forehead skin. No other measurements were described in this study.

### 3.2. Doppler Studies

#### 3.2.1. Central Artery

In both studies reporting a central artery, it was present in the superficial plane. It was found in 16 of 78 cases [4,22]. Cotofana et al. [22] observed the central artery in 24% of cases, and it was present in the superficial plane of the lower forehead. In the study by Phumyoo et al. [4], the central artery was detected in 4 of 28 hemifaces. At all landmarks, it was located mainly in the subcutaneous plane. The diameter of the CA was slightly larger, and the depth from the skin was greater in FHL0. The distance from the skin surface at various measurement points ranged from 0.6 to 3.8 mm and from the bone surface from 2.0 to 4.8 mm. The detailed measurement results are shown in Table 5 and Table 6.

#### 3.2.2. Paracentral Artery

Cotofana et al. [22] observed the paracentral artery in 20% of cases. No further details were described. Phumyoo et al. [4] detected the PCA in three hemifaces. In two cases, it was located in the subcutaneous plane in all landmarks. In one case, in FHL0, the artery was identified in the intra-orbicularis oculi muscle plane. As with CA, the diameter of the PCA was slightly larger, and the depth from the skin was greater in FHL0. The distance from the skin surface at different landmarks ranged from 1.4 to 6.0 mm and from the bone surface from 1.3 to 3.0 mm. The detailed results are shown in Table 5 and Table 6.

#### 3.2.3. Supratrochlear Artery

The supratrochlear artery was described in ten studies on ultrasound examination of the arteries [4,10,11,16,20,22,24,25,26,27]. It was detected in 547 of 555 cases. The distance from the midline was determined in three studies and ranged from 0 to 23 mm, depending on the article and measurement points [10,25,26]. However, it is worth mentioning that Cotofana et al. [10] noted no STrA between 0 and 5 mm, with occurrence primarily observed between 16 and 20 mm. Similarly, Shen et al. [26] reported that in almost all cases, the distance oscillated between 10 and 20 mm, while Schwenn et al. [25] detected the artery at a distance between 13 and 20 mm from the midline only. The position and distance from the glabellar wrinkles were also described in three studies [10,11,20]. In one, the pedicle of the supratrochlear artery was found lateral to the line of the glabellar wrinkles at a distance of 0 to 11 mm [20]. On the other hand, the lateral position was noted in 59% of cases in the study by Lee et al. [11], while in the remaining cases, the artery was detected at the line of the glabellar wrinkles. Unfortunately, detailed distance measurements were not performed. Cotofana et al. [10] reported that the distance from the glabellar wrinkles ranged from 0.9 to 19.0 mm but mostly oscillated between 2.9 and 19 mm. The depth of the STrA with respect to the skin surface was determined in four studies, while for the bone surface, it was specified in three articles [4,10,22,24]. Given the difference in measurement methods, detailed results for each study are presented below. In addition, two studies mentioned the point at which the artery became superficial. In the first study, which considered the deep branch, it was found to be 4–27 mm from the supraorbital margin. In the second study, the point at which the STrA became superficial was identified as the frontal prominence [16,22]. The diameter was given in five studies, and the mean values ranged from 0.4 to 1.03 mm, depending on the measurement point and branch (superficial or deep) [4,10,16,26,27].

In the study by Ugur et al. [20], the STrA pedicle was observed in 111 of 114 patients’ forehead sides. The distance from the MC varied from 0 to 3 mm, while the distance to the GFL ranged from 0 to 11 mm. The detailed results of the measurements are shown in Table 5. Significant differences in men and women were observed for the location of the SVP in relation to the medial canthus. The SVP was located medial to the medial canthus in 42 women and 6 men, while lateral localization was observed in 20 men and 5 women. As mentioned above in the study by Cotofana et al. [22], the distance between the supraorbital and supratrochlear arteries and the skin surface or the periosteum measured in the selected points were assessed. Details are shown in Table 5. Change in the location of the STrA from deep to superficial in relation to the frontalis muscle was also evaluated. The STrA as well as the SOA ran from deep to superficial to the frontalis muscle, and the change in course with respect to the superior orbital rim was observed at an average of 13.64 ± 2.3 mm (range 10.0–19.0) in men and an average of 13.67 ± 3.9 (range 4.0–27.0 mm) mm in women. This corresponded to 18.48% ± 3.3% and 20.30% ± 6.2% of the total forehead length, respectively, in men and women. The correlation of the distance of both the STrA and SOA from the skin surface and bone surface with age and sex were described earlier in this article. Güvenç et al. [24] observed the course of the STrA from its onset point on the eyebrow to a point 1.5 cm above the eyebrow. The authors observed four patterns in the artery’s course based on differences between the left and right arteries. These patterns include both arteries starting at the level of the eyebrow and extending 1.5 cm above it; the right artery originating at the eyebrow level and the left artery emerging from the right artery just above the eyebrow; the left artery starting at the eyebrow level and the right artery originating from the left artery above the eyebrow; and finally, both arteries starting at the eyebrow level, with the right artery continuing up to 1.5 cm above and the left artery ending just above the eyebrow. The authors also observed that the distance of the STrA from the bone surface ranged from 0.6 to 3.8 mm at a point 1.5 cm above the eyebrow and 0.7–3.7 mm at the level of the eyebrow. With respect to the skin surface, the distance was 1.8–5.9 mm at the eyebrow level and 1.8–5.1 mm at a point 1.5 cm above the eyebrow. The distance from the pericranium, measured at the eyebrow level, was greater in men. Moreover, there was a linear correlation between body mass index and the distance of the STrA from both the pericranium and the epidermis. Additionally, a positive correlation was noted between the distance of the STrA from the epidermis and age. The results of the measurements are shown in Table 6. Lee et al. [11] investigated the location of the STrA in relation to glabellar wrinkles. The artery was located laterally in 44 of the 74 cases, while in the remaining cases, it was found at the glabellar wrinkles. In addition, the authors evaluated whether the artery was located in the deep subcutaneous layer or subdermal layer. The latter location—the target for filler injection—was detected in 8% of the cases. Phumyoo et al. [4] found a superficial branch of the supratrochlear artery in 25 hemifaces. In 96% of cases, the branch crossed the corrugator supercilii muscle at the FHL0 point. It then entered the frontalis muscle and usually ran in the subcutaneous plane. A significant difference in the diameters of the superficial branches was found between men and women at the FHL1 point. The deep branch of the supratrochlear artery was observed in 19 hemifaces. At the FHL0 level, in 14 cases, it was located under the muscle, near the bone. It ran in an intramuscular or supraperiosteal plane. Detailed measurements are shown in Table 5 and Table 6. According to findings of Schwenn et al. [25], the supratrochlear artery’s exit point was located near the medial rim of the eyebrow, as the received Doppler signal was strongest at that site. The authors also assessed the distance between that point and the midline, and it ranged from 13 to 20 mm. In the study by Shen et al. [26], the STrA branched from the ophthalmic artery and in the orbital foramen ran at a distance of 10 to 20 mm from the midline in almost all cases and 10 to 15 mm in about 68%. It mostly maintained a similar distance (10 to 20 mm) at the superior brow margin and was located superficially to the frontalis muscle. Tansatit et al. [27] observed the STrA in 59 of 60 sides of volunteers’ faces as a branch of the ophthalmic artery. As mentioned earlier, certain data are shown in Table 6. In another study by Tansatit et al. [16], the STrA was observed in 59 of 60 cases. It started from the medial edge of the orbit running vertically before passing through the medial end of the brow and continuing towards the forehead. Concerning the depth of the artery, at the orbital rim, the STrA passed over the corrugator supercilii muscle, further bending to the surface of the frontal bone, and then ran along the superciliary arch in the submuscular plane of the orbicularis oculi muscle. Next, at the superciliary depression, it continued in the plane of frontalis muscle. It became subcutaneous at the frontal prominence. It was observed that in 12% of cases, the STrA arose more medially than usual on the side of the radix. Cotofana et al. [10] followed the run of the STrA and estimated its location depth as well as its distance from midline and vertical glabellar line both at rest and upon frowning. All measurements taken for the STrA are shown in Table 6. The authors observed that there was a zone 0–5 mm lateral of the midline where the artery was not found in any sample. Furthermore, it was found in the distance 16–20 mm most often. The course of the artery showed a statistically significant medial shift after maximum contraction of the glabella, regardless of the landmark, gender, or its initial position (medial or lateral to the landmark).

#### 3.2.4. Supraorbital Artery

The supraorbital artery was documented in six studies focusing on ultrasound investigation of the arteries [10,16,22,25,26,27]. It was detected in 236 of 238 cases. The distance from the midline was described in three studies with similar results [10,25,26]. The SOA was observed from 10 to 50 mm from the midline, depending on the study and measurement point. The most common distance was 16 to 35 or 20 to 40 mm, depending on the article. Two studies mentioned that the absence of SOA was detected at a distance of 0–10 mm from the midline [10,26]. The depth of the artery course from the skin surface was determined in two studies [10,22]. In the first, it was 2.2–6.0 mm as measured at the horizontal mid-eyebrow level, and in the second, it ranged from 1.68 to 3.67 mm regardless of location on the forehead. Moreover, in the latter study, the distance from the bone surface was also measured and was between 1.19 and 3.37 mm [22]. Two studies mentioned the point where the artery became superficial [16,22]. The first considered the deep branch, and it ranged from 4 to 24 mm as measured from supraorbital rim. In another, the point for the SOA was near the frontal prominence. The diameter was determined in four studies, and the average ranged from 0.70 to 0.79 mm [10,16,26,27].

Cotofana et al. [22] conducted an ultrasound investigation in which they evaluated the distance between the supratrochlear and supraorbital arteries and either skin surface or periosteum, measured at selected points corresponding to the proportion of the total forehead length (0, 20, 40, 60, and 100%). In addition, a change in the location of the arteries from deep to superficial in relation to the frontalis muscle was estimated. The distance between the SOA and the skin surface ranged from 1.68 to 3.67 mm, while the distance between the bone surface and the deep branch of the SOA varied from 1.19 to 3.37 mm. Details of the measurements are shown in Table 5. The deep branch of the SOA ran to the frontalis muscle to become superficial at an average of 13.32 ± 2.5 mm (range, 7.0–19.0 mm) and 14.10 ± 3.4 mm (range, 4.0–24.0 mm) from the superior orbital rim in men and women, respectively. This corresponded to points of total forehead length of 17.94% ± 2.9% in men and 20.91% ± 5.5% in women. In both the SOA and STrA, a correlation was observed between male gender and older age for an increased distance of the arteries from the skin surface. In contrast, in women, the distance was smaller with age. This was noted at all landmarks. The authors also noted that the distance between the arteries and bone surface in men increased with age, while it decreased in women. This was observed in the total length of the forehead from point 60% to 100%. Schwenn et al. [25], in their study conducted on 20 volunteers, observed the supraorbital arteries’ emerging points at the supraorbital rim and measured their distance from midline. Values ranged from 23 to 35 mm. The authors also identified the midportion of the supraorbital rim as a typical exit spot of that artery. In the study by Shen et al. [26], the SOA branched from the ophthalmic artery and, at the orbital foramen, ran mostly over 10 mm from the midline, primarily 20 to 40 mm (over 80% of cases) and 20 to 30 mm in more than 50% of cases. At the superior brow margin, the SOA ran superiorly and laterally, deep to the frontalis muscle, and at distances of 20 to 30 mm and 30 and 40 mm from the midline, the percentages were similar to those at the orbital foramen. Tansatit et al. [27] conducted an interesting study on the incidence of ophthalmic artery embolism occurring as an indirect result of an anastomosis between the external and internal carotid arteries. Although much of the data from the aforementioned study does not overlap with the area of interest in this review, there are some details of the STrA and SOA that could be extracted and are shown in Table 6. In another study by Tansatit et al. [16], the SOA was seen in 58 of 60 cases and ran lateral to the STrA. It started at the medial to middle brow between the vertical line of the medial limbus and the mid-pupillary vertical line. It then ran obliquely toward the temporal crest. At its starting point, the SOA was turning to the forehead, similar to the STrA. In 86%, it started as a branch of the superior orbitoglabellar artery, which crossed over the corrugator supercilii and became the SOA. In the remaining cases, it originated from the ophthalmic artery. The SOA ran near the orbital rim, deeper than STrA. It then passed through superciliary arch in the submuscular plane. At the superciliary depression, it crossed the frontalis muscle and ran to the frontal hairline. Near the frontal prominence, it coursed in the subcutaneous plane. In the study conducted by Cotofana et al. [10] on the course of supraorbital artery, the authors took measurements of the vessel, such as depth from skin surface and distance from both midline and vertical glabellar line, at rest and while contracting the forehead. The results are shown in Table 5. It was highlighted that in the distance between 0 to 10 mm lateral to the midline, there was no supraorbital artery reported in any case and that in most samples, it appeared in the distance 26–30 mm. According to the authors, only BMI was proven to significantly increase the depth of the artery’s course. The vessel also showed an explicit medial shift after frowning. This phenomenon occurred recurrently, regardless of the chosen landmark, gender, or position towards the landmark (medial or lateral).

### 3.3. CT Studies

#### 3.3.1. Central Artery

Koziej et al. [9] referred to the central artery in their article only to note that it was not detected. Instead, the authors mentioned the observation of a network of small vessels in the midline area in 34.7 cases.

#### 3.3.2. Paracentral Artery

The PCA was observed in 44.0% of cases in the Koziej et al. [9] study. The average diameter was 1.5 mm (Q1 = 1.2; Q3 = 1.9) and length 67.5 mm. It was the longest among all arteries. The PA was either a continuation of angular artery on the forehead (63.6%) or originated from the communicating branch with the STrA (36.4%). According to their findings and calculations concerning measured distances and both the 10th and 90th percentiles, the authors estimated that the paracentral artery should be expected 2–15 mm laterally to the midline.

#### 3.3.3. Supratrochlear Artery

The supratrochlear artery was described in five studies employing computed tomography and was observed in almost all cases [9,23,29,30,31]. Distinction of the superficial branch was conducted in three studies; usually, the superficial branch was observed in all cases, and in one study, it was noted in the forehead in only 20% of cases [29,30,31]. A deep branch was described in two studies and was observed in about 50% of cases [29,30].

In the study by Koziej et al. [9], the STrA was detected in 97.3% of cases. The median diameter was 1.4 mm (Q1: 1.2; Q3: 1.6), and the length was 53.2 mm. In 32.9%, the artery entered the forehead by notch and in 13.7% via the foramen. In the remaining cases, the authors observed no changes in the structure of the superior orbital rim. According to their findings and calculations concerning measured distances and both the 10th and 90th percentiles, the authors estimated that the STrA should be expected 11–21 mm laterally to the midline. They highlighted the fact that the STrA’s and PCA’s appearance zones did overlap. Liao et al. [29] distinguished between deep and superficial StrA and SOA. A superficial STrA (sSTrA) was detected in 100% of cases, while a deep STrA (dSTrA) was detected in only 52.1%. The starting point of the sSTrA was the superficial superior orbital arcade in 61.7% of cases, the ophthalmic artery in 24.5%, and the angular artery in the remaining cases. The dSTrA originated from the deep superior orbital arcade in 42.6% of cases and from the ophthalmic artery in 9.6% of hemifaces. The sSTrA was observed to run vertically, lateral to the midline, and at the lower medial edge of the orbital rim anastomosed to the frontal branch of the superficial temporal artery. The dSTrA ascended on the forehead to the frontal prominence near the periosteum. Zhao et al. [31] claimed that the STrA along with the SOA are the main sources of deep arteries in the forehead. According to their findings, in 90% of cases, the STrA originated from the trochlear branch of the ophthalmic arteries, in 7.5% from the angular artery, and in the other 2.5% from the supraorbital branch of the ophthalmic artery. The deep branch of the STrA originated from the trochlear branch of the ophthalmic artery at the superior part of the medial orbital rim and then ran upwards along the periosteum in 95% samples. Only in 5% of scanned samples was there a stage where the STrA showed a horizontal course running from supraorbital fissure along the orbital rim lying on the periosteum to the superior part of the medial orbital rim, where it turned upwards, along periosteum. The authors also observed the superficial branches of the STrA, which appeared in the forehead only in 20% of examined samples. All of them were from superior palpebral arteries. In their analysis of static CT scans, Reece et al. [23] observed that the supraorbital STrA, SOA, and dorsal nasal artery connected to form a supraorbital plexus. This plexus is believed to run from the nasal sidewall to the lateral orbital rim. Its superior margin reaches from 0 to 6.5 mm above the supraorbital rim. The STrA is said to run over the periosteum at the supraorbital rim and to branch into deep and superficial branches nearby. From that point, the deep branch ran consistently on the periosteum for 3 cm minimum, while the superficial branch rose up to the subcutaneous tissue as it was moving upwards. In another study by Liao et al. [30], the authors scanned 51 cadavers, receiving 3D computed tomography scans of 86 hemifaces in total. A superficial branch of the STrA was found in 100% of scanned hemifaces. The deep branch, on the other hand, appeared in only 53.5% of specimens.

#### 3.3.4. Supraorbital Artery

As with the STrA, the supraorbital artery was reported in five studies using CT scans and was observed in almost all cases [9,23,29,30,31]. The superficial branch was also described in three studies and was usually observed in all cases; in one study, the authors reported the sSOA in the forehead in 50% of cases [29,30,31]. The deep branch was described in two studies and was observed in all cases [29,30].

In the study by Koziej et al. [9], the supraorbital artery was noted in 89.3% of cases. The median diameter was 1.3 mm (Q1 = 1.0; Q3 = 1.7), and length was 39.2 mm. It entered the forehead either by notch (67.2%) or foramen in the remaining cases. According to their findings and calculations concerning measured distances and both the 10th and 90th percentiles, the authors estimated that the SOA should be expected 21–32 mm laterally to the midline. In the study by Liao et al. [29], both the sSOA and dSOA were detected in 100% of hemifaces. The sSOA emerged at the lower medial margin of the orbital rim, while the dSOA emerged near the supraorbital foramen or notch, and both vessels continued upward and lateral, supplying the lateral part of the forehead. In 94.7% of cases, the sSOA originated from the superficial superior orbital arcade and in the remaining cases from the OA. The sSOA originated from the deep superior orbital arcade in 42.6%, from the OA in 43.6%, and from the deep superior orbital artery in the remaining cases. In another study by the aforementioned authors [30], both superficial and deep branches of the supraorbital artery were also found in all examined samples. Zhao et al. [31] noted that the SOA in 65% ran along periosteum on the distance between superior part of the medial orbital rim and supraorbital fissure and later on taking an upward course along the periosteum. In all remaining cases (35%), it appeared at the supraorbital fissure and took an upward course along the periosteum. Only 50% of SOAs were shown to have superficial branches, all of them originating from the superior palpebral artery. Reece et al. [23] counted the SOA as a component of the supraorbital plexus described previously in STrA descriptions. The analysis of acquired dynamic CT scans has brought authors to the conclusion that SOA supplies the supraorbital territory mainly through the plexus.

#### 3.3.5. Frontal Branch of the Superficial Temporal Artery

Hong et al. [3] in their study split the course of the FBSTA into two stages. The first stage describes the artery before it crossed the temporal crest, which was common for both anatomical variants of FBSTA. After the arterial trunk passed the temporal crest, the authors observed there were two main variants of the remaining part and named them type 1 FBSTA and type 2 FBSTA. In the type 1 FBSTA, which concerns the majority of examined cases, the vessel turned upwards along the temporal crest and ascended towards the hairline. In the other variant (10.28%), the main trunk’s turn at this point was gentler, and the vessel supplied the upper lateral part of the forehead. The authors also measured the distance from the supraorbital rim to the crossing point of FBSTA and temporal crest, as shown in Table 7; however, the values did not vary significantly between the variants. Koziej et al. [28], by means of computed tomography angiography and 3-dimensional volume-rendering postprocessing of images, acquired reconstructed models of the superficial temporal artery and its main branches: the frontal and parietal ones. The authors decided to measure the diameter of the FBSTA at points located 1 cm and 7 cm distally to the branching point of STA to estimate the vertical distance between FBSTA and several landmarks, such as the center of the supraorbital margin, lateral angle of the orbital rim, and corpus of the zygomatic bone. The mean diameter was 0.97 ± 0.32 (0.4–2.3) 1 cm distal to the branching point and 0.81 ± 0.26 (0.3–2.4) 7 cm distal to it. All other measurements listed above are shown in Table 7. In the article, it is emphasized that there is a strong correlation between the location of the branching point of STA and values such as distance from FBSTA to the lateral angle of the orbital rim or the corpus of the zygomatic bone and the angle between FBSTA and PBSTA. The correlation manifests as an increase in these values as the branching point goes upwards.

## 4. Discussion

In recent years, there has been an increase in the popularity of aesthetic dermatology procedures, reflecting the growing interest in improving the appearance and rejuvenation of the skin [2,14] The area of the face with one of the greatest aesthetic significances due to its prominence, proximity to the eyes, and rapidly appearing signs of aging is the forehead [30,31]. Knowledge of the vascular system of the forehead is crucial for dermatological procedures performed in this region, especially fillers or autologous fat injection [4,24,32]. Studies have shown that the glabella region is one of the most dangerous zones for these procedures due to the rich distribution of arteries and vascular variability [4,17,33]. During procedures in this area, ocular complications are most commonly encountered [33,34]. To assess the topography of arteries, researchers employ various methods, such as cadaver dissection, ultrasonography, and computed tomography. Naturally, all of these methods have their advantages and disadvantages. For example, dissection is the least accurate for determining the depth of arteries due to the removal of overlying soft tissue [16]. While ultrasound may be less precise, it is more suitable for determining depth within the surrounding soft tissues [4]. Detection of arteries less than 0.3 mm in diameter may not be possible on some Doppler ultrasound equipment. Moreover, the primary advantage of ultrasound is its ability to provide real-time results and its applicability to many procedures. In addition, it is non-invasive, safe, and inexpensive [35]. Computed tomography provides quick and easy arterial information without the need for dissection, yet it still permits high-throughput study of vascular variation [31]. However, it requires a minimum arterial lumen diameter (about 0.4 mm), and smaller vessels may not be visualized. Additionally, there is a risk of obtaining unclear images in some cases.

The current review focuses on arteries crucial for the blood supply to the forehead [9]. These arteries are observed over a relatively wide area, and their locations vary. Additionally, the branches of these vessels may follow different courses and run through different layers of forehead tissue, and specific branches or even entire arteries may be absent in some individuals.

The central artery runs medially to the central forehead, while the paracentral artery runs laterally to the CA [4]. These vessels are visible in an area ranging from 0.2 to 10.8 mm for the CA and 0.8 to 16.2 mm for the PCA on the entire path from the glabellar point to the frontal prominence point. Despite their importance in the blood supply to the central forehead, these arteries seem to be overlooked in anatomy textbooks [9]. These vessels also hold significance for the risk of procedures. In the Cotofana et al. [22] study, the authors noted that the CA and PCA were observed superficially in the superficial fat layer in 24% and 20% of cases, respectively. These arteries contribute to a network of vessels in the glabella region connected to the angular and ophthalmic arteries. For this reason, the authors cautioned against injecting fillers into the superficial fat layer due to the inconsistency and unpredictability of these superficial vessels. Cong et al. [8] divided the distribution patterns of the central forehead arteries into two types based on the presence of the deep branch of the supratrochlear artery. It was not present in all cases, in contrast to the superficial branches of the STrA and SOA and the deep branch of the SOA. Interestingly, the CA and PCA were observed in 20% of cases but only in the type of distribution where the deep branch of the STrA was present. Koziej et al. [9] suggested that the PCA may be another vessel besides the STrA and SOA that can cause ophthalmic complications due to occlusion of the ophthalmic artery. In the case of the PCA, this may be due to its connection to the angular or supratrochlear artery.

The supratrochlear and supraorbital arteries were present in most cases in this review. Typically, they gave off both superficial and deep branching, although some studies described additional branching patterns. In the included studies, these arteries were measured at various landmarks and observed within relatively wide zones. The STrA and SOA have been measured at different landmarks in numerous cadaver studies, with some reporting results for the superficial and deep branches separately. The distances from the midline at various measurement points ranged from 0.6 to 28.0 mm for the superficial branch of the STrA [4]. For the deep branch of the STrA, the distance from the midline ranged from 13.6 to 40.7 mm [4]. In case of the SOA, the distance from the midline ranged from 23 to 32 mm [21,25]. In ultrasonographic studies, the STrA and SOA were detectable in most cases. Measurements from the midline ranged from 0 to 23 mm, with the majority falling between 10 and 20 mm for the former artery [10,25,26]. For the SOA, the distance from the midline ranged from 10 to 50 mm, with the most common occurrence between 20 and 40 mm [10,25,26]. It is worth noting that in no study was the artery observed between 0 and 10 mm from the midline, which may suggest some safe zones for this vessel. In studies employing computed tomography, the STrA and the SOA were detected in almost all cases. Koziej et al. reported that the STrA was observed at a distance of 11 to 21 mm and the SOA at a distance of 21 to 32 mm, both lateral to the midline [9]. This is consistent with reports from studies using other methods of arterial evaluation. Considering both of these vessels, it becomes apparent that the arteries are situated between 0 and 50 mm from the midline. This seems particularly important because the mentioned arteries are frequently discussed concerning the safety of aesthetic procedures, precisely because of their connection to the ophthalmic artery. The injection of filler into these arteries can lead to material entering the ophthalmic artery, potentially causing vision impairment, including blindness [36].

Interestingly, some studies took measurements from the glabellar frown line (GFL). For the STrA, the distance ranged between 0 and 19.0 mm and for SOA between 6.2 and 31.1 mm. This landmark seems to be important especially in the context of the STrA. It was even suggested that the GFL may be a reliable landmark for the STrA [37]. In Lee et al.’s [11] study, 41% of STrAs were located just at the GFL. A study by Vural [37] reported that a superficial branch of the STrA was located directly below the frown in 49% of cases. In contrast, Cotofana et al. [10] reported that in 98% of cases, the STrA was located at a distance greater than 1 mm from the GFL. Therefore, the authors suggested that the hypothesis of the safety of injection next to the GFL due to the location of the artery in the GFL should be rejected. The same authors also highlighted another crucial aspect: The soft tissues of the glabella are mobile. They described variations in arterial localization during muscle contraction. This is another factor that makes the glabella area particularly dangerous with regard to the aesthetic procedures. The authors even suggested that neuromodulators such as botulinum toxin should be administered first to make the location of the artery more predictable on ultrasound.

Another important consideration is the change in the location of the artery (whether superficial or deep). This aspect was addressed in two ultrasound studies [16,22]. In one study, measurements from the supraorbital rim ranged from 4 to 24 mm for the SOA and 4 to 27 mm for the STrA [22]. In the other study, it was the point of the frontal prominence for both arteries [16]. Some studies suggest a division based on this change in arterial location, distinguishing between the upper forehead (considered safer for fillers injected into the supraperiosteal plane) and the less safe lower forehead [38,39]. Concerns about injecting deeply into the lower forehead arise from potential complications such as vision problems [39]. However, findings from the study by Cotofana et al. [22] indicated that deep injection into the lower part of the forehead may not carry such a high risk, as both arteries were still not in direct contact with the periosteum. The distance from the periosteum was approximately 1.19–3.37 mm for the SOA and 1.09–3.29 mm for the STrA. Moreover, Hong et al. [3] observed more superficial than deep arteries in the midline area, suggesting that the space above the periosteum may be safer for filler injections than the subcutaneous layer due to the lower incidence of arteries. The authors recommended the injection of fillers in between the frontalis and periosteum. Based on their findings, Phumyoo et al. [4] also recommended injecting fillers into the periosteal layer rather than the subcutaneous tissue layer, as it is more challenging to avoid arteries in the latter. Guvenc et al. [24] also seemed to support these findings, reporting a distance from the bone surface for the STrA at the level of the eyebrow of 0.7–3.7 mm and 1.5 cm above the level of the eyebrow of 0.6–3.8 mm. However, based on their findings, the authors recommended not injecting deeper than 1.8 mm, as they observed that the distance from the epidermis to the artery at the level of the eyebrow ranged from 1.8 to 5.9 mm and, similarly, at 1.5 cm above the level of the eyebrow ranged from 1.8 to 5.1 mm.

The other artery evaluated was the frontal branch of the superficial temporal artery (FBSTA), described in two cadaveric and two CT studies and observed in most cases. This artery may have some relevance in the context of procedures safety. Cotofana and Lachman [39] found that the FBSTA connects to a branch of the supraorbital artery. Nevertheless, Tansatit et al. [27] reported that in about two-thirds of the subjects, there was no effective connection of the FBSTA to the terminal branches of the ophthalmic artery, indicating that the risk of blindness due to FBSTA cannulation is low. The authors, however, did not rule out this possibility.

A summary of the selected measurements is shown in Table 8.

## 5. Conclusions

The multitude of variations in arteries and their occurrence across a relatively wide area and at different depths highlights the importance for medical professionals to be aware of zones where forehead arteries are more likely to be encountered. The glabella region seems to be one of the most dangerous zones for dermatological procedures. Its rich vascularization and soft tissue mobility, resulting in changes in arterial localization during muscle contraction, increase the risk of vascular complications. In addition to the supratrochlear and supraorbital arteries, the paracentral artery is considered another vessel that can cause ophthalmic complications due to occlusion of the ophthalmic artery. The risk of ophthalmic complications associated with occlusion of the frontal branch of the superficial temporal artery appears to be low but cannot be entirely ruled out. In comparison to the periosteal layer, the subcutaneous tissue layer appears to be more challenging for avoiding arteries.

## Figures and Tables

**Table 1:** Characteristics of the studies included in the review.

References	Location	Number of Group (Male, Female, Race)	Age (Years)	Arteries Evaluated	Method of Arteria Evaluation
Phumyoo et al., 2019 [4]	Thailand	38 hemifaces (11 males, 8 females; Asian)	55–90	Central artery, paracentral artery, supratrochlear artery	Cadaver dissection
		14 volunteers (3 males, 11 females; Asian), 28 hemifaces	20–41	Central artery, paracentral artery, supratrochlear artery	Ultrasonographic investigation
Cong et al. 2017 [8]	Korea, Thailand	20 hemifaces of 11 cadavers (7 males, 4 females; Asian)	mean 74.5	Supraorbital artery, supratrochlear artery	Cadaver dissection
Jo et al., 2012 [13]	Korea	10 hemifaces of 6 cadavers (3 males, 3 females; Asian)	66–86	Frontal branch of the superficial temporal artery	Cadaver dissection
Kleintjes, 2007 [14]	South Africa	60 hemiforeheads (30 cadavers)	n-a	Frontal branch of superficial temporal artery, supratrochlear artery, supraorbital artery, central artery, paracentral artery	Cadaver dissection
Pinar and Govsa, 2006 [15]	Turkey	14 cadavers (13 males, 1 female), 27 hemifaces	43–75	Frontal branch of superficial temporal artery	Cadaver dissection
Tansatit et al., 2019 [16]	Thailand	20 cadavers, 40 hemifaces	54–69	Supraorbital artery, supratrochlear artery	Cadaver dissection
		30 volunteers, 60 hemifaces	22–45	Supraorbital artery, supratrochlear artery	Ultrasonographic investigation
Khan et al., 2016 [17]	USA	6 cadavers (5 males, 1 female; Caucasian and African-American), 12 hemifaces	n-a	Supratrochlear artery	Cadaver dissection
Erdogmus and Govsa, 2007 [18]	Turkey	19 males cadavers (38 orbits; Asian), 38 hemifaces	43–75	Supraorbital artery	Cadaver dissection
Yu et al., 2010 [19]	China	10 cadavers (9 males, 1 female)	65–78	Supratrochlear artery	Cadaver dissection
Ugur et al., 2008 [20]	Turkey	57 volunteers (23 males, 34 females), 114 hemifaces	18–62	Supratrochlear artery	Ultrasonographic investigation
		15 cadavers	n-a	supratrochlear artery	Cadaver dissection
Palomar-Gallego et al., 2019 [21]	Spain	17 hemifaces cadavers (11 males, 6 females)	53–79	Supratrochlear artery, supraorbital artery	Cadaver dissection
Cotofana et al., 2021 [22]	USA	50 patients (11 males, 39 females; Caucasian and Asian)	20–79	Supraorbital artery, supratrochlear artery	Ultrasonographic investigation
Reece et al., 2008 [23]	USA	5 cadavers (4 males, 1 female)	55–97	Supratrochlear artery	Cadaver dissection
				Supraorbital artery, supratrochlear artery	Computed tomography
Güvenç et al., 2022 [24]	Turkey	71 patients (29 males, 42 females)	16–72	Supratrochlear artery	Ultrasonographic investigation
Schwenn et al., 2005 [25]	Germany	12 cadavers, 12 hemifaces	n-a	Supraorbital artery, supratrochlear artery	Cadaver dissection
		20 patients (8 males, 12 females), 40 orbits	24–42	Supraorbital artery, supratrochlear artery	Ultrasonographic investigation
Lee et al., 2021 [11]	Korea	42 patients (4 males, 38 females), 74 hemifaces	38–71	Supratrochlear artery	Ultrasonographic investigation
Shen et al., 2022 [26]	China	37 patients (14 males, 23 females; Asian)	20–49	Supraorbital artery, supratrochlear artery	Ultrasonographic investigation
Tansatit et al., 2018 [27]	Thailand	30 volunteers (17 males, 13 females), 60 hemifaces	22–45	supraorbital artery, supratrochlear artery	Ultrasonographic investigation
Cotofana et al., 2020 [10]	USA	41 volunteers (20 males, 21 females; Caucasian and African-American), 82 hemifaces	20–56	Supraorbital artery, supratrochlear artery	Ultrasonographic investigation
Koziej et al., 2020 [9]	Poland	40 cadavers, 75 hemispheres (34 males, 6 females)	19–79	Supraorbital artery, supratrochlear artery, paracentral artery	Computed tomography
Koziej et al., 2018 [28]	Poland	215 patients (89 males, 126 females; Caucasian)	18–92	Frontal branch of superficial temporal artery	Computed tomography
Liao et al., 2022 [29]	China	56 cadavers, 94 hemifaces (34 males, 22 females; Asian)	18–71	Supraorbital artery, supratrochlear artery	Computed tomography
Liao et al., 2020 [30]	China	51 cadavers, 86 hemifaces (20 males, 31 females; Asian)	28–62	Supraorbital artery, supratrochlear artery	Computed tomography
Zhao et al., 2018 [31]	China	21 cadavers, 40 hemifaces (7 males, 14 females; Asian)	23–48	Supraorbital artery, supratrochlear artery	Computed tomography
Hong et al., 2020 [3]	China	59 cadavers (Asian), 107 hemifaces	n-a	Frontal branch of the superficial temporal artery	Computed tomography

n-a—non-acquired.

**Table 2:** Landmarks utilized, arterial evaluation methods, and evaluated parameters.

References	Landmarks	Visualization of Arterial Distribution	Measurement of Arterial Parameters
Phumyo et al., 2020 [4]	*Y*-axis: vertical line at the midline of the face *X*-axis: three horizontal lines: 1. Line passing the supraorbital rim (FHL0: glabellar point) 2. Line passing the midpoint between the frontal prominence and FHL0 (FHL1: mid-frontal depression point) 3. Line passing the frontal prominence (FHL2: frontal prominence point)	Dissection; injection of red latex	Distances from the landmarks, diameter and depth of the arteries
		Real-time color Doppler ultrasound	The arteries courses from radix to central forehead, the diameters and depths of the arteries
Cong et al., 2017 [8]	X1—a horizontal line running from the medial canthus to the lateral canthus (through the palpebral fissure) X2—horizontal line running through the supraorbital margin Y—vertical line running through the medial canthus X SOF—horizontal distance from Y to the supraorbital foramen on the line X2 Y SOF—vertical height of the supraorbital foramen from X1 X dSOA—average horizontal distance of the deep branch of supraorbital artery penetration point on the frontalis from Y Y dSOA—average vertical height of the deep branch of the supraorbital artery penetration point on the frontalis from X2	Dissection; injection of red and blue latex	Distances of the supraorbital artery with respect to the landmarks
Jo et al., 2012 [13]	-	Dissection; injection of red latex or methylene blue solution	-
Kleintjes, 2007 [14]	STT—superior transverse third MTT—middle transverse third ITT—inferior transverse third LORV—lateral orbital rim vertical line MCV—medial canthal vertical line MCH—medial canthal horizontal line MCL—medial corneal limbus vertical line	Dissection; yellow, red, or blue latex solution injection	Distances from the landmarks diameter of the arteries
Pinar and Govsa, 2006 [15]	-	Dissection; injection of red latex solution	Course and diameter of the artery
Tansatit et al., 2019 [16]	-	Dissection; injection of red latex	Courses of the arteries
		Doppler ultrasound	Courses of the arteries, diameter and depths of the arteries
Khan et al., 2017 [17]	-	Dissection; injection of red latex	Length, radius, and volume of vessel lumen
Erdogmus and Govsa, 2007 [18]	-	Dissection; injection of colored latex	The origin point, location, and course of the arteries
Yu et al., 2010 [19]	Midline, supraorbital rim	Dissection; injection of red latex	The distances from the offset point of the cutaneous branch of StrA to the midline and supraorbital rim, diameter
Ugur et al., 2008 [20]	MC—vertical line passing through the medial canthus GFL—glabellar frown line	Doppler ultrasound	Measurements conducted at the level of superior border of the medial brow: - The distance between the supratrochlear vascular pedicle (SVP) GFL - The distance between SVP and MC - The orientation (lateral or medial) of the SVP to GFL and MC
	MC	Dissection	The distance between SVP and MC
Palomar-Gallego et al., 2019 [21]	Medial palpebral commissure Glabellar midline	Dissection; injection of red latex	Distances from the landmarks
Cotofana et al., 2021 [22]	Length of the forehead—mean vertical distance between the upper margin of the eyebrow and the frontal hairline Six landmarks created by dividing the length of the forehead into five equal segments: 0%, 20%, 40%, 60%, 80%, and 100% of total forehead length	Doppler ultrasound	Distances between: - Skin surface and periosteum - Skin surface and the arteries - The arteries and periosteum Change in the location of the arteries from deep to superficial in relation to the frontalis muscle
Güvenç et al., 2022 [24]	Eyebrow Epidermis Periosteum	Doppler ultrasound	Depth of artery: - At the level of eyebrow - 1.5 cm above the eyebrow Distance from the upper wall of the artery to the epidermis (EAD) Distance from the lower wall of the artery to the periosteum (APD)
Reece et al., 2008 [23]	Orbital rim	Dissection; injection of red latex	Distance of the superficial branch of the supratrochlear artery from the orbital rim
		Computed tomography	Distance from the supraorbital rim, the course of arterial plexus
Schwenn et al., 2005 [25]	Midline	Cadaver dissection; injection of India ink	Distance between the vessels leaving orbital rim and the midline Diameter
	Midline	Doppler ultrasonography	Distance between the vessels leaving orbital rim and the midline
Lee et al., 2021 [11]	Glabella wrinkles Deep subcutaneous layer Subdermal layer	Doppler ultrasonography	Location of STrA in relation to the landmarks
Shen et al., 2022 [26]	Midline Orbital foramen Superior brow margin	Doppler ultrasonography	Distance from the midline Diameter
Tansatit et al., 2018 [27]	-	Doppler ultrasonography	Diameter of the arteries
Cotofana et al., 2020 [10]	Midline Vertical glabellar line	Doppler ultrasonography	Measurements at the horizontal mid-eyebrow level: - Diameters - Distance between skin surface and the arteries at rest and upon frowning - Distance between the midline and the arteries at rest and upon frowning - Distance between vertical glabellar lines and the arteries at rest and upon frowning
Koziej et al., 2020 [9]	Central point—The cross point of the horizontal line passes through the summits of the superior orbital rims and the midline - The horizontal distance between the central point and the crossing point of the: A—paracentral artery B—supratrochlear artery C—supraorbital artery with the supraorbital rim D—the horizontal distance between supraorbital and supratrochlear arteries - Vertical extension of the: E—paracentral artery F—supratrochlear artery G—supraorbital artery - The distance between the central point and the crossing point of the: H—supratrochlear artery I—supraorbital artery with the supraorbital rim - The distance from the center of the medial orbital rim to the crossing point of the: J—supratrochlear artery K—supraorbital artery with the supraorbital rim	Computed tomography with administration of oily liquid contrast medium: 6% Angiofil paraffin oil solution Three-dimensional volume rendered reconstructions	-Lengths of arteries -Distances form the landmarks
Koziej et al., 2018 [28]	- Bifurcation point—described in relation to the central point of zygomatic arch: “+”—up above “_”—below the point - Supraorbital margin - The lateral angle of the orbital rim - The corpus of zygomatic bone	Computed tomography with nonionic contrast agent injection Three-dimensional volume-rendered reconstructions	Frontal branch of superficial temporal artery: - Diameter 1 cm and 7 cm above STA bifurcation - Distances from landmarks
Liao et al., 2022 [29]	-	Computed tomography with mix of 40 g of lead oxide, 5 mL of red dye, and 100 mL of latex	Location and course of the arteries Branching
Liao et al., 2020 [30]	-	Computed tomography with mix of 40 g of lead oxide, 5 mL of red dye, and 100 mL of latex	Branching
Zhao et al., 2018 [31]	-	Computed tomography with mix of 40 g of lead oxide, 5 mL of red dye, and 100 mL of latex	Location and course of the arteries Branching
Hong et al., 2020 [3]	Supraorbital rim	Computed tomography with mix of 40 g of lead oxide, 5 mL of red dye, and 100 mL of latex	Angle between horizontal line and the main trunk of the artery Distance between the supraorbital rim and the artery

**Table 3:** Mean diameters (in mm) of the arteries in cadaveric studies measured at the different orientation points.

Author	Central Artery	Paracentral Artery	Supratrochlear Artery	Supraorbital Artery	Frontal Branch of STA
Kleintjes, 2007 [14]	<1	<1	1	1	2
Pinar and Govsa, 2006 [15]	-	-	-	-	2.14 ± 0.54
Khan et al., 2017 [17]	-	-	1.44	-	-
Schwenn et al., 2005 [25]	-	-	1.08 ± 0.19 (0.8–1.5)	0.86 ± 0.19 (0.6–1.2)	-
Phumyoo et al., 2019 [4]	0.7 ± 0.3 (0.4–1.2)	0.6 ± 0.1 (0.4–0.7)	Superficial branch: 0.7 ± 0.2 (0.3–1.1) Deep branch: 0.6 ± 0.1 (0.3–1.0)	-	-

**Table 4:** Mean distance (range) from landmarks in millimeters (mm) in cadaveric studies.

Author	Landmark	Central Artery	Paracentral Artery	Supratrochlear Artery		Supraorbital Artery
				Superficial br.	Deep br.	
Phumyoo et al., 2019 [4]	At origin point: X—distance from midline Y—distance from vertical line passing through the supraorbital rim at the mid-pupillary point	X: 4.7 ± 1.8 (1.4–7.4) Y: −13.2 ± 2.7 (−17.7–−8.0)	X: 9.4 ± 3.1 (2.7–12.5) Y: −13.9 ± 4.7 (−19.4–−7.4)	X: 15.0 ± 3.2 (8.0–23.5) Y: 6.7 ± 2.7 (−12.1–0)	X: 18.5 ± 2.5 (13.6–22.2) Y: 0.6 ± 1.2 (−2.8–−0.1)	-
	At penetration point: X—distance from midline Y—distance from vertical line passing through the supraorbital rim at the mid-pupillary point	-	-	X: 13.9 ± 3.3 (5.2–19.4) Y: 7.6 ± 2.6 (0–15.1)	X: 25.5 ± 5.6 (18.1–33.3) Y: 30.2 ± 5.1 (23.6–35.7)	-
	Glabellar point (FHL0)	4.7 ± 2.3 (0.2–7.8)	7.8 ± 2.4 (4.6–11.5)	14.7 ± 3.2 (6.6–21.7)	19.2 ± 1.6 (16.8–22.1)	-
	FHL1	4.4 ± 2.0 (0.7–10.5)	8.8 ± 2.7 (0.8–16.2)	12.2 ± 3.1 (4.0–24.4)	21.4 ± 3.9 (23.6–35.7)	-
	FHL2	3.6 ± 2.6 (0.7–10.8)	9.5 ± 3.9 (1.3–14.3)	11.7 ± 4.2 (0.6–28.0)	27.2 ± 6.1 (18.2–40.7)	-
Cong et al, 2017 [8]	Y horizontally	-	-	-	-	Penetration point: 29.6 ± 4.1 Supraorbital foramen: 10.8 ± 4.9
	X2 vertically	-	-	-	-	Penetration point: 20.7 ± 5.1
	X1 vertically	-	-	-	-	Supraorbital foramen: 22.1 ± 2.6
Ugur et al., 2008 [20]	MC	-	-	Total SVP to MC: 0.7 ± 0.6 Lateral to MC: 1.2 ± 0.4 (0–3) Medial to MC: 1.0 ± 0.0 (0–2) At MC: 0.0		-
Palomar-Gallego et al., 2019 [21]	MC	-	-	Origin point: 11.5 (8–13.5)		-
	Glabellar midline	-	-	-		Origin point: 25 (23–27)
Schwenn et al., 2005 [25]	Midline at the supraorbital rim	-	-	16.4 ± 2.2 (range 14–21)		27.2 ± 2.8 (23–32)
Yu et al., 2010 [19]	Midline			13.5 ± 3.4		
	Supraorbital rim			11.8 ± 3.6		
Erdogmus and Govsa, 2007 [18]	Supraorbital rim					Penetration point: 20–40

**Table 5:** Distance from landmarks in millimeters (mm) in ultrasonography studies.

Author	Landmark		Central Artery	Paracentral Artery	Supratrochlear Artery		Supraorbital Artery	
					Superficial br.	Deep br.	Superficial br.	Deep br.
Ugur et al., 2008 [20]	MC		-	-	At MC: 0.00 Lateral: 1.2 ± 0.4 (0–3) Medial: 1.2 ± 0.5 (0–2)		-	
	GFL		-	-	6.2 ± 1.7 (0 to 11)		-	
Cotofana et al., 2021 [22]	Skin surface	0%	-	-	-	Men: 3.40 ± 0.8 Women: 3.57 ± 1.0	-	Men: 3.92 ± 1.3 Women: 3.80 ± 1.1
		20%	-	-	-	Men: 2.94 ± 0.6 Women: 2.85 ± 0.6	-	Men: 2.80 ± 0.5 Women: 2.88 ± 0.8
		40%	-	-	-	Men: 2.67 ± 0.6 Women: 2.57 ± 0.6	-	Men: 2.76 ± 0.5 Women: 2.60 ± 0.6
		60%	-	-	-	Men: 2.69 ± 0.6 Women: 2.39 ± 0.6	-	Men: 2.68 ± 0.5 Women: 2.52 ± 0.6
		80%	-	-	-	Men: 2.90 ± 1.6 Women: 2.33 ± 0.5	-	Men: 2.62 ± 0.4 Women: 2.38 ± 0.5
		100%	-	-	-	Men: 2.38 ± 0.5 Women: 2.25 ± 0.6	-	Men: 2.53 ± 0.6 Women: 2.28 ± 0.5
	Periosteum	0%	-	-	-	Men: 2.50 ± 1.0 Women: 2.38 ± 1.0	-	Men: 2.36 ± 0.6 Women: 2.34 ± 1.2
		20%	-	-	-	Men: 2.42 ± 0.6 Women: 2.16 ± 0.6	-	Men: 2.54 ± 0.7 Women: 2.06 ± 0.6
		40%	-	-	-	Men: 2.55 ± 0.7 Women: 2.21 ± 0.6	-	Men: 2.38 ± 0.6 Women: 2.23 ± 0.6
		60%	-	-	-	Men: 2.40 ± 0.5 Women: 2.18 ± 0.6	-	Men: 2.59 ± 0.7 Women: 2.10 ± 0.7
		80%	-	-	-	Men: 2.37 ± 0.7 Women: 2.01 ± 0.7	-	Men: 2.43 ± 0.7 Women: 2.17 ± 0.7
		100%	-	-	-	Men: 2.32 ± 0.7 Women: 2.02 ± 0.6	-	Men: 2.43 ± 0.7 Women: 2.15 ± 0.7
Güvenç et al., 2022 [24]	EAD at the eyebrow level		-	-	Right artery: 3.61 ± 0.75 (2.2–5.9) Left artery: 3.47 ± 0.64 (1.8–5.6)		-	
	APD at the eyebrow level		-	-	Right artery: 1.86 ± 0.59 (0.9–3.7) Left artery: 1.85 ± 0.58 (0.7–3.1)		-	
	EAD 1.5 cm above eyebrow		-	-	Right artery: 3.25 ± 0.73 (1.8–4.7) Left artery: 3.27 ± 0.71 (2.0–5.1)		-	
	APD 1.5 cm above eyebrow		-	-	Right artery: 1.66 ± 0.58 (0.7–3.5) Left artery: 1.58 ± 0.64 (0.6–3.8)		-	
Phumyoo et al., 2019 [4]	FHL0	Depth from the skin	3.1 ± 0.7 (2.0–3.8)	4.8 ± 1.7 2.8–6.0)	4.2 ± 0.8 (2.6–6.1)	5.9 ± 1.6 (3.3–10.0)	-	
		Depth to the bone	2.6 ± 0.4 (2.0–2.86)	2.1 ± 0.7 (1.3–2.9)	2.9 ± 0.9 (0.5–4.3)	2.0 ± 1.4 (0.5–4.76)	-	
	FHL1	Depth from the skin	2.2 ± 1.0 (1.3–3.8)	2.0 ± 0.6 (1.4–2.8)	2.1 ± 0.9 (0.6–3.8)	3.8 ± 1.5 (1.5–7.1)	-	
		Depth to the bone	2.8 ± 1.3 (1.9–4.8)	3.0 ± 0.2 (2.9–3.3)	2.6 ± 1.1 (0.9–5.0)	1.5 ± 0.6 (0.5–2.9)	-	
	FHL2	Depth from the skin	0.8 ± 0.2 (0.6–0.9)	1.8 ± 0.2 (1.7–1.9)	1.7 ± 0.5 (0.5–2.9)	2.5 ± 0.7 (1.4–4.1)	-	
		Depth to the bone	3.1 ± 1.6 (2.0–4.3)	2.3 ± 0.6 (1.9–2.8)	2.1 ± 0.5 (1.4–3.3)	1.6 ± 0.5 (0.7–2.4)	-	
Schwenn et al., 2005 [25]	Midline		-	-	16.4 ± 1.7 (13–20)		26.5 ± 2.6 (23–35)	
Shen et al., 2022 [26]	Midline (at the orbital foramen)		-	-	Right artery: 13.55 ± 2.53 (0–20) Left artery: 13.91 ± 2.52 (0–20)		Right artery: 25.81 ± 6.50 (10–50) Left artery: 25.48 ± 5.70 (10–50)	
	Midline (at the superior brow margin)		-	-	Right artery: 13.13 ± 3.37 (0–20) Left artery: 14.12 ± 3.40 (0–20)		Right artery: 28.20 ± 7.18 (10–50) Left artery: 29.10 ± 8.93 (10–50)	
Cotofana et al. 2020 [10]	Mid-eyebrow level	Skin surface at rest	-	-	3.34 ± 0.6 (1.8–4.6)		3.54 ± 0.8 (2.2–6.0)	
		Skin surface upon frowning	-	-	3.77 ± 0.7 (2.1–5.4)		3.93 ± 1.0 (1.8–6.3)	
	Midline	At rest	-	-	Men: 16.13 ± 3.8 (6.0–23.0) Women: 14.80 ± 3.5 (6.0–21.0)		Men: 27.93 ± 5.3 (15.3–36.9) Women: 27.31 ± 5.9 (13.8–39.1)	
		Upon frowning	-	-	Men: 13.27 ± 3.3 (5.4–19.7) Women: 11.53 ± 2.7 (4.4–15.5)		Men: 24.08 ± 5.5 (8.8–31.8) Women: 23.44 ± 5.4 (14.0–32.2)	
	GFL	At rest	-	-	Men: 10.59 ± 4.0 (2.9–19.0) Women: 8.21 ± 4.0 (–3.3–14.2)		Men: 22.38 ± 5.5 (6.8–31.1) Women: 20.73 ± 5.6 (6.2–28.8)	
		Upon frowning	-	-	Men: 8.96 ± 3.5 (2.3–17.0) Women: 6.37 ± 3.3 (–2.8–12.1)		Men: 19.76 ± 5.6 (2.8–29.1) Women: 18.28 ± 5.4 (7.1–27.2)	

**Table 6:** Mean diameters (range) of the arteries in ultrasonography studies (in mm) measured at the different orientation points.

Author	Landmark	Central Artery	Paracentral Artery	Supratrochlear Artery		Supraorbital Artery
				Superficial br.	Deep br.	
Phumyoo et al., 2019 [4]	FHL0	0.7 ± 0.3 (0.4–1.2)	0.6 ± 0.1 (0.4–0.7)	0.7 ± 0.2 (0.3–1.1)	0.6 ± 0.1 (0.3–1.0)	-
	FHL1	0.4 ± 0.1 (0.3–0.5)	0.4 ± 0.1 (0.3–0.5)	0.5 ± 0.2 (0.3–1.0)	0.5 ± 0.2 (0.3–1.0)	-
	FHL2	0.3 ± 0.1 (0.3–0.4)	0.4 ± 0.1 (0.3–0.4)	0.4 ± 0.1 (0.3–0.7)	0.6 ± 0.2 (0.3–1.0)	-
Shen et al., 2022 [26]	Midline (at the orbital foramen)	-	-	Right artery: 0.96 ± 0.24 Left artery: 1.03 ± 0.22		Right artery: 0.79 ± 0.25 Left artery: 0.78 ± 0.24
	Midline (at the superior brow margin)	-	-	Right artery: 0.78 ± 0.17 Left artery: 0.77 ± 0.15		Right artery: 0.73 ± 0.16 Left artery: 0.64 ± 0.14
Tansatit et al., 2018 [27]	-	-	-	0.80 ± 0.38		0.71 ± 0.25
Tansatit et al., 2019 [16]	-	-	-	0.80 ± 0.38		0.71 ± 0.25
Cotofana et al. 2020 [10]	Horizontal mid-eyebrow level			0.90 ± 0.02 (0.5–1.3)		0.70 ± 0.2 (0.5–1.4)

**Table 7:** Distance from landmarks in millimeters (mm) in CT studies.

Author	Landmark	Paracentral Artery	Supratrochlear Artery		Supraorbital Artery	Frontal Branch of the Superficial Temporal Artery
			Superficial br.	Deep br.		
Koziej et al., 2020 [9]	The horizontal distance between the central point and the crossing point of the artery with the supraorbital rim	10th percentile: 1.8 90th percentile: 15.4 Median: 7.6	10th percentile: 10.7 90th percentile: 20.6 Median: 15.1		10th percentile: 20.7 90th percentile: 31.8 Median: 26.5	-
	The horizontal distance between SOA and STrA	-	10th percentile: 5.0 90th percentile: 14.9 Median: 9.2			-
	Vertical extension of the artery	10th percentile: 30.2 90th percentile: 73.6 Median: 62.6	10th percentile: 21.4 90th percentile: 71.2 Median: 48.1		10th percentile: 20.6 90th percentile: 60.9 Median: 35.8	-
	The distance between the central point and the crossing point of the artery with the supraorbital rim	-	10th percentile: 12.9 90th percentile: 20.6 Median: 17.4		10th percentile: 20.3 90th percentile: 31.3 Median: 26.5	-
	The distance from the center of the medial orbital rim to the crossing point of the artery with the supraorbital rim	-	10th percentile: 11.5 90th percentile: 18.6 Median: 14.4		10th percentile: 18.4 90th percentile: 25.6 Median: 22.4	-
Koziej et al., 2018 [28]	Bifurcation point—in relation to the central point of zygomatic arch (ZA)	-	-		-	Above ZA: n = 282 +23.8 ± 11.4 (2.6–65.3) Below ZA: n = 55 −8.1 ± 4.4 (−23.4–2.3) at ZA: n = 36
	Vertical distance between the center of the supraorbital margin and the FBSTA	-	-		-	10th percentile: 47.3 90th percentile: 84.0 Median: 70.4
	Vertical distance between the lateral angle of the orbital rim and the FBSTA	-	-		-	10th percentile: 23.0 90th percentile: 55.9 Median: 36.6
	Vertical distance between the point of the corpus of zygomatic bone (between the frontal and temporal processes) and the FBSTA	-	-		-	10th percentile: 25.7 90th percentile: 54.9 Median: 42.5
Hong et al., 2020 [3]	Distance between the supraorbital rim and the artery	-	-		-	31.58 ± 8.33

**Table 8:** Summary of selected measurement ranges for each artery based on data from various studies.

Artery	Landmark (at Various Measurement Points)	Range of Measurement (mm)	Method of Measurement	Citation
Central artery	Midline	0.2 to 10.8	Cadaveric dissection	[4]
	Bone surface	2.0 to 4.8	Ultrasonography	[4]
	Skin surface	0.6 to 3.8	Ultrasonography	[4]
Paracentral artery	Midline	0.8 to 16.2	Cadaveric dissection	[4]
	Bone surface	1.3 to 3.0	Ultrasonography	[4]
	Skin surface	1.4 to 6.0	Ultrasonography	[4]
Supratrochlear artery	Midline	0.6 to 40.7	Cadaveric dissection	[4,25]
		11 to 21	Computed tomography	[9]
		0 to 23	Ultrasonography	[10,25,26]
	Glabellar frown Line	0 to 19.0	Ultrasonography	[10,20]
	Bone surface	0.5 to 5.0	Ultrasonography	[4,22,24]
	Skin surface	0.5 to 10	Ultrasonography	[4,10,22,24]
Supraorbital artery	Midline	23 to 32	Cadaveric dissection	[21,25]
		10 to 50	Ultrasonography	[10,25,26]
		21 to 32	Computed tomography	[9]
	Glabellar frown line	6.2 to 31.1	Ultrasonography	[10]
	Bone surface	1.19 to 3.37	Ultrasonography	[22]
	Skin surface	1.68 to 3.67	Ultrasonography	[10,22]

## Data Availability

Not applicable.

## Supplementary Materials

The following supporting information can be downloaded at: https://www.mdpi.com/article/10.3390/jcm13144238/s1, Supplementary Material File S1: Search terms used in the review.
