# Supplementary material for: Arterial Vascularization of the Forehead in Aesthetic Dermatology Procedures: A Review
Source: J Clin Med. 2024 Jul 19;13(14):4238. doi: 10.3390/jcm13144238 (PMC11278280; doi:10.3390/jcm13144238)
Supplement: Supplementary file 1 [file jcm-13-04238-s001.zip › jcm-3093064-supplementary.pdf]

Supplementary Material File S1: Search terms used in the review

Pubmed:

(forehead arteries AND filler) OR (forehead arteries AND filler injections) OR (forehead arteries AND aesthetic procedures) OR (forehead arteries AND dermatology) OR (arteries of the frontal region AND filler injections) OR (arteries of the frontal region AND aesthetic procedures) OR (arteries of the frontal region AND dermatology) OR (forehead arteries AND fillers) OR (forehead arteries AND fillers injections) OR (forehead arteries AND aesthetic medicine) OR (forehead arteries AND aesthetic dermatology) OR (forehead arteries AND cosmetic dermatology) OR (forehead arteries AND cosmetic procedures) OR (arteries of the forehead AND cosmetic dermatology) OR (arteries of the forehead AND filler) OR (arteries of the forehead AND filler injections) OR (arteries of the forehead AND aesthetic dermatology) OR (arteries of the forehead AND aesthetic medicine) OR (arteries of the frontal region AND filler) OR (arteries of the frontal region AND fillers) OR (arteries of the frontal region AND aesthetic medicine) OR (arteries of the frontal region AND aesthetic dermatology) OR (forehead arteries AND dermatosurgery) OR (forehead arteries AND dermatologic surgery) OR (arteries of the frontal region AND fillers injections) OR (arteries of the frontal region AND cosmetic procedures) OR (arteries of the frontal region AND cosmetic dermatology) OR (arteries of the frontal region AND dermatosurgery) OR (arteries of the frontal region AND dermatologic surgery) OR (arteries of the forehead AND fillers) OR (arteries of the forehead AND fillers injections) OR (arteries of the forehead AND dermatology) OR (arteries of the forehead AND aesthetic procedures) OR (arteries of the forehead AND cosmetic procedures) OR (arteries of the forehead AND dermatosurgery) OR (arteries of the forehead AND dermatologic surgery)

Embase:

('forehead'/exp OR forehead) AND ('arteries'/exp OR arteries) AND ('filler'/exp OR filler) OR (('forehead'/exp OR forehead) AND ('arteries'/exp OR arteries) AND ('filler'/exp OR filler) AND ('injections'/exp OR injections)) OR (('forehead'/exp OR forehead) AND ('arteries'/exp OR arteries) AND aesthetic AND ('procedures'/exp OR procedures)) OR (('forehead'/exp OR forehead) AND ('arteries'/exp OR arteries) AND ('dermatology'/exp OR dermatology)) OR (('arteries'/exp OR arteries) AND of AND the AND frontal AND region AND ('filler'/exp OR filler) AND ('injections'/exp OR injections)) OR (('arteries'/exp OR arteries) AND of AND the AND frontal AND region AND aesthetic AND ('procedures'/exp OR procedures)) OR (('arteries'/exp OR arteries) AND of AND the AND frontal AND region AND ('dermatology'/exp OR dermatology)) OR (('forehead'/exp OR forehead) AND ('arteries'/exp OR arteries) AND fillers) OR (('forehead'/exp OR forehead) AND ('arteries'/exp OR arteries) AND fillers AND ('injections'/exp OR injections)) OR (('forehead'/exp OR forehead) AND ('arteries'/exp OR arteries) AND aesthetic AND ('medicine'/exp OR medicine)) OR (('forehead'/exp OR forehead) AND ('arteries'/exp OR arteries) AND aesthetic AND ('dermatology'/exp OR dermatology)) OR

(('forehead'/exp OR forehead) AND ('arteries'/exp OR arteries) AND ('cosmetic'/exp OR cosmetic) AND ('dermatology'/exp OR dermatology)) OR (('forehead'/exp OR forehead) AND ('arteries'/exp OR arteries) AND ('cosmetic'/exp OR cosmetic) AND ('procedures'/exp OR procedures)) OR (('arteries'/exp OR arteries) AND of AND the AND ('forehead'/exp OR forehead) AND ('cosmetic'/exp OR cosmetic) AND ('dermatology'/exp OR dermatology)) OR (('arteries'/exp OR arteries) AND of AND the AND ('forehead'/exp OR forehead) AND ('filler'/exp OR filler)) OR (('arteries'/exp OR arteries) AND of AND the AND ('forehead'/exp OR forehead) AND ('filler'/exp OR filler) AND ('injections'/exp OR injections)) OR (('arteries'/exp OR arteries) AND of AND the AND ('forehead'/exp OR forehead) AND aesthetic AND ('dermatology'/exp OR dermatology)) OR (('arteries'/exp OR arteries) AND of AND the AND ('forehead'/exp OR forehead) AND aesthetic AND ('medicine'/exp OR medicine)) OR (('arteries'/exp OR arteries) AND of AND the AND frontal AND region AND ('filler'/exp OR filler)) OR (('arteries'/exp OR arteries) AND of AND the AND frontal AND region AND fillers) OR (('arteries'/exp OR arteries) AND of AND the AND frontal AND region AND aesthetic AND ('medicine'/exp OR medicine)) OR (('arteries'/exp OR arteries) AND of AND the AND frontal AND region AND aesthetic AND ('dermatology'/exp OR dermatology)) OR (('forehead'/exp OR forehead) AND ('arteries'/exp OR arteries) AND ('dermatosurgery'/exp OR dermatosurgery)) OR (('forehead'/exp OR forehead) AND ('arteries'/exp OR arteries) AND dermatologic AND ('surgery'/exp OR surgery)) OR (('arteries'/exp OR arteries) AND of AND the AND frontal AND region AND fillers AND ('injections'/exp OR injections)) OR (('arteries'/exp OR arteries) AND of AND the AND frontal AND region AND ('cosmetic'/exp OR cosmetic) AND ('procedures'/exp OR procedures)) OR (('arteries'/exp OR arteries) AND of AND the AND frontal AND region AND ('cosmetic'/exp OR cosmetic) AND ('dermatology'/exp OR dermatology)) OR (('arteries'/exp OR arteries) AND of AND the AND frontal AND region AND ('dermatosurgery'/exp OR dermatosurgery)) OR (('arteries'/exp OR arteries) AND of AND the AND frontal AND region AND dermatologic AND ('surgery'/exp OR surgery)) OR (('arteries'/exp OR arteries) AND of AND the AND ('forehead'/exp OR forehead) AND fillers) OR (('arteries'/exp OR arteries) AND of AND the AND ('forehead'/exp OR forehead) AND fillers AND ('injections'/exp OR injections)) OR (('arteries'/exp OR arteries) AND of AND the AND ('forehead'/exp OR forehead) AND ('dermatology'/exp OR dermatology)) OR (('arteries'/exp OR arteries) AND of AND the AND ('forehead'/exp OR forehead) AND aesthetic AND ('procedures'/exp OR procedures)) OR (('arteries'/exp OR arteries) AND of AND the AND ('forehead'/exp OR forehead) AND ('cosmetic'/exp OR cosmetic) AND ('procedures'/exp OR procedures)) OR (('arteries'/exp OR arteries) AND of AND the AND ('forehead'/exp OR forehead) AND ('dermatosurgery'/exp OR dermatosurgery)) OR (('arteries'/exp OR arteries) AND of AND the AND ('forehead'/exp OR forehead) AND dermatologic AND ('surgery'/exp OR surgery))

Web of science:

TS=((forehead AND arteries AND filler) OR (forehead AND arteries AND filler AND injections) OR (forehead AND arteries AND aesthetic AND procedures) OR (forehead AND arteries AND dermatology) OR (arteries AND of AND the AND frontal AND region AND filler AND injections) OR (arteries AND of AND the AND frontal AND region AND aesthetic AND procedures) OR (arteries

AND of AND the AND frontal AND region AND dermatology) OR (forehead AND arteries AND fillers) OR (forehead AND arteries AND fillers AND injections) OR (forehead AND arteries AND aesthetic AND medicine) OR (forehead AND arteries AND aesthetic AND dermatology) OR (forehead AND arteries AND cosmetic AND dermatology) OR (forehead AND arteries AND cosmetic AND procedures) OR (arteries AND of AND the AND forehead AND cosmetic AND dermatology) OR (arteries AND of AND the AND forehead AND filler) OR (arteries AND of AND the AND forehead AND filler AND injections) OR (arteries AND of AND the AND forehead AND aesthetic AND dermatology) OR (arteries AND of AND the AND forehead AND aesthetic AND medicine) OR (arteries AND of AND the AND frontal AND region AND filler) OR (arteries AND of AND the AND frontal AND region AND fillers) OR (arteries AND of AND the AND frontal AND region AND aesthetic AND medicine) OR (arteries AND of AND the AND frontal AND region AND aesthetic AND dermatology) OR (forehead AND arteries AND dermatosurgery) OR (forehead AND arteries AND dermatologic AND surgery) OR (arteries AND of AND the AND frontal AND region AND fillers AND injections) OR (arteries AND of AND the AND frontal AND region AND cosmetic AND procedures) OR (arteries AND of AND the AND frontal AND region AND cosmetic AND dermatology) OR (arteries AND of AND the AND frontal AND region AND dermatosurgery) OR (arteries AND of AND the AND frontal AND region AND dermatologic AND surgery) OR (arteries AND of AND the AND forehead AND fillers) OR (arteries AND of AND the AND forehead AND fillers AND injections) OR (arteries AND of AND the AND forehead AND dermatology) OR (arteries AND of AND the AND forehead AND aesthetic AND procedures) OR (arteries AND of AND the AND forehead AND cosmetic AND procedures) OR (arteries AND of AND the AND forehead AND dermatosurgery) OR (arteries AND of AND the AND forehead AND dermatologic AND surgery))

Scopus:

TITLE-ABS-KEY(forehead arteries AND filler) OR TITLE-ABS-KEY(forehead arteries AND filler injections) OR TITLE-ABS-KEY(forehead arteries AND aesthetic procedures) OR TITLE-ABS-KEY(forehead arteries AND dermatology) OR TITLE-ABS-KEY(arteries of the frontal region AND filler injections) OR TITLE-ABS-KEY(arteries of the frontal region AND aesthetic procedures) OR TITLE-ABS-KEY(arteries of the frontal region AND dermatology) OR TITLE-ABS-KEY(forehead arteries AND fillers) OR TITLE-ABS-KEY(forehead arteries AND fillers injections) OR TITLE-ABS-KEY(forehead arteries AND aesthetic medicine) OR TITLE-ABS-KEY(forehead arteries AND aesthetic dermatology) OR TITLE-ABS-KEY(forehead arteries AND cosmetic dermatology) OR TITLE-ABS-KEY(forehead arteries AND cosmetic procedures) OR TITLE-ABS-KEY(arteries of the forehead AND cosmetic dermatology) OR TITLE-ABS-KEY(arteries of the forehead AND filler) OR TITLE-ABS-

KEY(arteries of the forehead AND filler injections) OR TITLE-ABS-KEY(arteries of the forehead AND aesthetic dermatology) OR TITLE-ABS-KEY(arteries of the forehead AND aesthetic medicine) OR TITLE-ABS-KEY(arteries of the frontal region AND filler) OR TITLE-ABS-KEY(arteries of the frontal region AND fillers) OR TITLE-ABS-KEY(arteries of the frontal region AND aesthetic medicine) OR TITLE-ABS-KEY(arteries of the frontal region AND aesthetic dermatology) OR TITLE-ABS-KEY(forehead arteries AND dermatosurgery) OR TITLE-ABS-KEY(forehead arteries AND dermatologic surgery) OR TITLE-ABS-KEY(arteries of the frontal region AND fillers injections) OR TITLE-ABS-KEY(arteries of the frontal region AND cosmetic procedures) OR TITLE-ABS-KEY(arteries of the frontal region AND cosmetic dermatology) OR TITLE-ABS-KEY(arteries of the frontal region AND dermatosurgery) OR TITLE-ABS-KEY(arteries of the frontal region AND dermatologic surgery) OR TITLE-ABS-KEY(arteries of the forehead AND fillers) OR TITLE-ABS-KEY(arteries of the forehead AND fillers injections) OR TITLE-ABS-KEY(arteries of the forehead AND dermatology) OR TITLE-ABS-KEY(arteries of the forehead AND aesthetic procedures) OR TITLE-ABS-KEY(arteries of the forehead AND cosmetic procedures) OR TITLE-ABS-KEY(arteries of the forehead AND dermatosurgery) OR TITLE-ABS-KEY(arteries of the forehead AND dermatologic surgery)
